# QuickStats

**Published:** 2014-05-23

**Authors:** FROM THE NATIONAL CENTER FOR HEALTH STATISTICS

**Figure:**
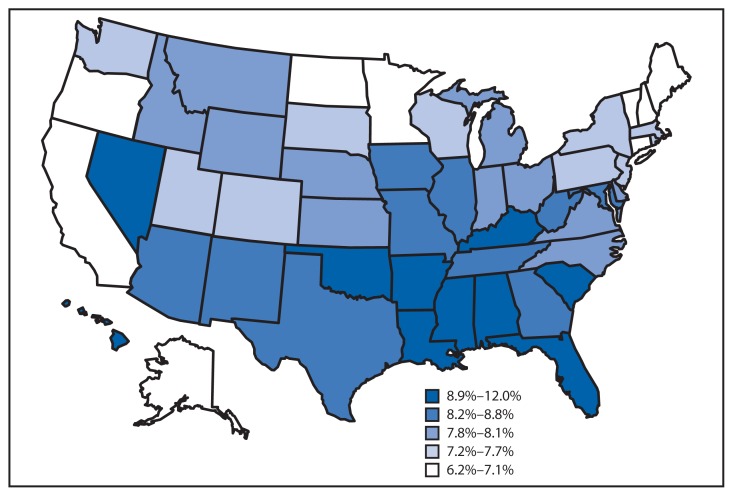
Percentage of Infants Born Late Preterm,* by Mother’s State of Residence — National Vital Statistics System, United States, 2012 * Defined as a gestational age (interval between the date of the mother’s last normal menses and the date of birth) of 34–36 completed weeks. Late preterm births accounted for 70% of all preterm births in 2012 and are considered to be at less risk than births at <34 weeks (early preterm) but at greater risk for birth complications and subsequent health or medical problems than full-term births.

In 2012, 8.1% of births in the United States were late preterm births. The percentage of births that were late preterm varied by state and ranged from 6.2% in Vermont to 12.0% in Mississippi.

**Sources:** Martin JA, Hamilton BE, Osterman, MJK, Curtin SC, Mathews T. Births: final data for 2012. Natl Vital Stat Rep 2013;62(9). Available at http://www.cdc.gov/nchs/data/nvsr/nvsr62/nvsr62_09.pdf.

US Department of Health and Human Services. Health indicators warehouse. Hyattsville, MD: US Department of Health and Human Services; 2013. Available at http://www.healthindicators.gov.

**Reported by:** Kate M. Brett, PhD, kbrett@cdc.gov, 301-458-4113; Li-Hui Chen, PhD.

